# Model-informed experimental design recommendations for distinguishing intrinsic and acquired targeted therapeutic resistance in head and neck cancer

**DOI:** 10.1038/s41540-022-00244-7

**Published:** 2022-09-08

**Authors:** Santiago D. Cárdenas, Constance J. Reznik, Ruchira Ranaweera, Feifei Song, Christine H. Chung, Elana J. Fertig, Jana L. Gevertz

**Affiliations:** 1 Department of Mathematics and Statistics, The College of New Jersey, Ewing, NJ USA; 2 Department of Head and Neck-Endocrine Oncology, Moffitt Cancer Center, Tampa, FL USA; 3 Convergence Institute, Department of Oncology, Department of Biomedical Engineering, Department of Applied Mathematics and Statistics, Johns Hopkins University, Baltimore, MD USA; 4 Present Address: Datacor, Inc., Florham Park, NJ USA

**Keywords:** Cancer, Computational biology and bioinformatics

## Abstract

The promise of precision medicine has been limited by the pervasive resistance to many targeted therapies for cancer. Inferring the timing (i.e., pre-existing or acquired) and mechanism (i.e., drug-induced) of such resistance is crucial for designing effective new therapeutics. This paper studies cetuximab resistance in head and neck squamous cell carcinoma (HNSCC) using tumor volume data obtained from patient-derived tumor xenografts. We ask if resistance mechanisms can be determined from this data alone, and if not, what data would be needed to deduce the underlying mode(s) of resistance. To answer these questions, we propose a family of mathematical models, with each member of the family assuming a different timing and mechanism of resistance. We present a method for fitting these models to individual volumetric data, and utilize model selection and parameter sensitivity analyses to ask: which member(s) of the family of models best describes HNSCC response to cetuximab, and what does that tell us about the timing and mechanisms driving resistance? We find that along with time-course volumetric data to a single dose of cetuximab, the initial resistance fraction and, in some instances, dose escalation volumetric data are required to distinguish among the family of models and thereby infer the mechanisms of resistance. These findings can inform future experimental design so that we can best leverage the synergy of wet laboratory experimentation and mathematical modeling in the study of novel targeted cancer therapeutics.

## Introduction

In cancer, each individual’s tumor has undergone a distinct set of molecular and cellular alterations that promote malignancy. Advances to high-throughput measurement technologies have enabled unprecedented characterization of these alterations, ushering in a new era of precision medicine which selects therapies to target the specific changes in each tumor. In spite of the promise of these precision medicine strategies, many cancers do not respond as anticipated to such targeted therapeutic strategies, and those who do respond frequently develop resistance.

Head and neck squamous cell carcinoma (HNSCC) is the sixth most common cancer worldwide with a 5-year survival rate of 50%^1^. Increased expression of the epidermal growth factor receptor (EGFR) occurs in 90% of HNSCC and is associated with poor survival^2,3^. EGFR is a receptor in certain types of cells that binds to epidermal growth factors, which are involved in cell signaling pathways controlling cell division and survival. Therefore, targeted therapeutics inhibiting EGFR have been developed to block these pathways as a precision therapeutic to prevent cancer cells from growing. Cetuximab is the only targeted therapy FDA approved for HNSCC^4^. Including cetuximab as part of an advanced stage HNSCC treatment plan exhibits a survival advantage for the patient compared to radiation treatment alone. Cetuximab also improves response rates compared to chemotherapy in patients with metastatic or recurrent HNSCC^5^. However, only a subset of patients are intrinsically sensitive to cetuximab, and responsive patients will develop resistance within 1–2 years^4,6–8^. The widespread prevalence of cetuximab resistance is currently limiting its clinical utility in HNSCC.

There are three different types of drug-resistance to consider in understanding resistance to targeted therapies such as cetuximab: pre-existing, randomly-acquired, and drug-induced acquired. Pre-existing resistance is when all resistance in the tumor population exists before treatment begins. Treatment then selects for these resistant cells, giving rise to a resistant tumor. Random acquired resistance occurs when resistant cells arise during treatment due to random genetic mutations or phenotypic switching, but not as a result of the drug administered. Cells for which resistance is pre-existing or randomly acquired act as a substrate for Darwinian evolution^9^. Lastly, drug-induced resistance is resistance directly caused by the drug during treatment, either through genetic changes or more likely through non-genetic cell phenotype plasticity^9–12^. These cells, often called drug-resistant or drug-tolerant persisters, act as a substrate for Lamarckian evolution as the adaptive changes occur as a direct response to the drug itself^9^. Our previous molecular profiling studies of HNSCC cell lines suggest that compensatory growth factor signaling and epithelial to mesenchymal transition (EMT) associated with cetuximab resistance is seen early in treatment^13^, though cannot distinguish if this resistance was pre-existing or acquired. Further, the precise contribution of growth factor signaling and EMT to subsequent resistance requires further longitudinal profiling which can be confounded by evolutionary processes in culture^14^ and infeasible to extend to powered, temporal profiling in in vivo models.

Mathematical models have been widely utilized to help understand drug resistance, and its consequences for treatment response and design—see ref. ^15–18^ for reviews of modeling work on cancer drug resistance. Overwhelmingly, these models have assumed that resistance is either pre-existing (as in ref. ^19–22^), or is a combination of pre-existing and spontaneously acquired resistance (as in ref. ^23–25^). More recently, modeling has also considered the contribution of the drug itself in driving the formation of resistance. Works such as ref. ^11,26–29^ consider drug-induced resistance, though they are limited in their ability to make predictions regarding doses and dosages that differ from the data used to validate them, as these models are dose-independent. A handful of mathematical models have been developed in which resistance is induced by the drug itself in a dose-dependent fashion^30–33^. The modeling family herein is strongly motivated by the single model proposed in ref. ^33^, wherein pre-existing, spontaneously acquired, and dose-dependent drug-induced resistance are modeled through a minimal system of two ordinary differential equations.

In this work, we propose a family of mathematical models, with each “member” of the family assuming a different timing and mechanism of cetuximab resistance. In “Methods”, we detail the protocol for collecting the experimental data, describe the family of mathematical models (where each member of the family represents a different set of mechanisms driving resistance), explain the algorithm for fitting these models to individual volumetric data, and introduce the methodology for assessing parameter sensitivity/identifiability. In “Results”, we employ information criteria (IC) to try and identify the most parsimonious model to describe the data. Extending such an information theoretic approach to our family of resistance models allowed us to confidently conclude that the data cannot be explained without resistance, and that the combination of pre-existing and randomly acquired resistance is very unlikely to be the mechanism responsible for the resistance to cetuximab observed in the experiments. In “Results”, we use a profile likelihood analysis to demonstrate that single-cell experiments which measure the resistance fraction in the initial tumor population provide powerful data for selecting the model (and therefore the underlying mechanisms) most parsimonious with the experimental data. In the case where this measure of pre-existing resistance does not allow the mechanism of resistance to be definitively determined using our family of models, we further propose that a dose-escalation experiment would provide the needed data to identify the model whose mechanisms best-explain cetuximab resistance. The “Discussion” contains closing remarks and reflections about the role mathematical modeling can play in experimental design to decipher the mechanism(s) of resistance to targeted therapeutics.

## Methods

### Experimental data

In this work, we utilize tumor volume data obtained from temporally monitoring a cetuximab responsive patient-derived tumor xenograft HNSCC model. Tumor tissue were collected from surgically resected HNSCC patients under the auspices of a tissue bank protocol approved by Johns Hopkins University Institutional Review Board. All animal studies and care were approved by the Institutional Animal Care and Use Committee of the Johns Hopkins University and Moffitt Cancer Center. Following HNSCC tumor resection, de-identified patient samples were implanted into athymic nude mice (Crl: NU-Foxn1nu, 4–6 weeks old; 20 g; Harlan Laboratories, Indianapolis, IN) and passaged to subsequent generations of mice for expansion. The mice are then divided into two groups: the control group and the treatment group. For each group, tumor volume is tracked over time under the assumption that $V=l{w}^{2}\frac{\pi }{6}$, where *l* is length and *w* is width of the tumor. Treatment (either with a placebo, or with cetuximab) starts when the tumor volume is ~200 mm^3^. Mice were euthanized if tumor volume surpassed ~2000 mm^3^, if they lost more than 25% of their body weight, or if ulceration occurred on the skin over the tumors. Batches of mice from each group were also euthanized at routine time points to enable temporal profiling for additional studies.

The control data is obtained by administering a weekly dose of phosphate-buffered saline (PBS) to tumor-bearing mice. We classified each control mouse into one of three categories: increasing volume, decreasing volume, and stabilized volume. Out of 25 control mice, 19 show increasing volume (see Mouse 23 in Fig. 2), one shows decreasing volume (see Mouse 11 in Fig. 2), and five show stabilization (see Mouse 22 in Fig. 2).

The treatment data is obtained by following the same procedure as the control mice, except that mice were given a 5 mg/kg intraperitoneal injection of cetuximab once every 7 days. As with the control mice, each mouse was classified as either increasing in volume (treatment failure), decreasing in volume (treatment success), or stabilized volume. Out of 29 mice, 19 show increasing volume (see Mouse 13 in Fig. 4), seven show decreasing volume (see Mouse 23 in Fig. 4), and three show stabilization (see Mouse 24 in Fig. 4). Despite the variation seen across individual mice in the control and treatment group, a Fisher’s exact test has a 2-sided *p*-value of 0.06 of decreased tumor volume occurring in the treatment group relative to control group. This suggests a trend towards cetuximab response in this xenograft model.

To account for outliers and noise in our data, we applied a censor to remove any data points deemed not biologically plausible. Estimates from literature of doubling time for HNSCC vary widely, from 26 h in culture to 44 days in vivo^34,35^. When fitting an exponential model (which assumes a constant cell doubling time) to each of the control mice, the fastest cell-doubling time we observed across these mice was 13 days. We took a conservative approach to censoring the data: in any case where the data show the tumor more than doubling in volume in 3–4 days (meaning, the volume doubles significantly faster than anything we observed in the control data), and the subsequent time points are not consistent with that rapid doubling time (meaning, the larger increase in volume is not sustained beyond that one point), we remove the outlier volume. As an example, if *V*(*t*_1_) = *V*_1_ and *V*(*t*_2_) = 6*V*_1_, we would censor the volume at time *t* = *t*_2_ if *V*(*t*_3_) < < 6*V*_1_ (meaning the growth seen at time *t*_2_ was not sustained). Otherwise, we would not censor the *t*_2_ data point. We show two examples to depict our censoring approach in Supplementary Fig. 2, one with a censored point due to an unsustained rapid doubling, and one without censoring despite a rapid doubling as it was sustained beyond that time point. In the control data, exactly one data point was removed from five of 25 mice, and in the treatment data nine data points were censored across seven of 29 mice.

### Modeling control data

Before building a model of tumor growth in response to treatment, we first considered how to best-describe tumor growth in the absence of treatment. There are a multitude of mathematical equations to describe tumor growth, and the equation chosen can have important consequences on model predictions^36–38^. Herein, we considered three different models of tumor growth: exponential, logistic, and Allee. These models were chosen because they represent a hierarchy of complexity.

Exponential growth simply assumes the growth rate of the tumor volume *V* is proportional to the tumor volume:

1 $$\frac{dV}{dt}=rV.$$

Logistic growth adds a rate-limiting factor to uncontrolled exponential growth, accounting for environmental constraints on tumor progression through a carrying capacity *K*:

2 $$\frac{dV}{dt}=rV\left(1-\frac{V}{K}\right).$$

Finally, the Allee effect further adds the assumption that the growth rate can also be limited by a population size that is below the Allee threshold *m*:

3 $$\frac{dV}{dt}=rV\left(1-\frac{V}{K}\right)\left(\frac{V}{m}-1\right).$$

There are multiple plausible explanations for why tumor growth could be described by such a differential equation. It may be that tumors grow slower because of uptake challenges in the xenograft system, or because they have yet to accumulate significant mutations. As an example, the growth kinetics of BT-474 luminal B breast cancer cells was shown to be best-described by a model structure that considers the Allee effect^39^.

All models considered herein are empirical models, meaning they do not directly incorporate the mechanisms that underlie tumor growth. There is an abundance of literature that aims to mechanistically describe tumor growth using systems of ordinary differential equations (for instance, in ref. ^40–43^), partial differential equations (for instance, in ref. ^44,45^), lattice/agent-based models (for instance, in ref. ^46–48^), and multi-scale models (for instance, in ref. ^49–51^). An excellent review of the range of model-based approaches for understanding tumor growth dynamics can be found in ref. ^52^. Many of these models explicitly incorporate spatial effects and reveal the important role space can play in tumor progression and response to treatment (see for instance^44–48,53,54^). Such mechanistic models can provide outstanding insight into tumor progression, but the control data available here did not allow such detailed models to be parameterized. Hence we restrict ourselves to consider more simplistic and empirical ordinary differential equation models.

### Modeling treatment data

Once the most parsimonious control model is selected, we can move to build a model that incorporates treatment response to cetuximab. We will use the following general modeling framework, where *S* is the volume of cells that are sensitive to cetuximab, *R* is the volume of cells with some level of resistance to cetuximab, and *D* is the concentration of drug:

4 $$\frac{dS}{dt}=(sensitive\,growth)-(transition\,to\,resistant)-(drug\,{{\mbox{-}}}\,induced\,sensitive\,death)$$

5 $$\frac{dR}{dt}=(resistant\,growth)+(transition\,to\,resistant)-(drug\,{{\mbox{-}}}\,induced\,resistant\,death)$$

6 $$\frac{dD}{dt}=-(decay).$$

We assume that growth is exponential (justified in “Selecting a Model: Control Data”), that the death rate is proportional to the drug concentration and the volume of the subpopulation, and that in Eq. (6) the only dynamics modeled are the natural decay of the drug. This simplifies our general modeling framework to have the form:

7 $$\frac{dS}{dt}={r}_{S}S-f(S,D)-{\lambda }_{S}DS$$

8 $$\frac{dR}{dt}={r}_{R}R+f(S,D)-{\lambda }_{R}DR$$

9 $$\frac{dD}{dt}=-\gamma D,$$

where *r*_*S*_ is the growth rate for sensitive cells, and *r*_*R*_ is the growth rate for resistant cells. We assume that resistance will either not impact the growth rate of cells, or it will result in a fitness disadvantage (*r*_*S*_ ≥ *r*_*R*_)^55,56^, though we note that more recently it has been observed that not all resistance comes at a cost^57^. *λ*_*S*_ is the drug-induced death term for sensitive cells, and *λ*_*R*_ is the drug-induced death term for resistant cells. We assume that *λ*_*S*_ > *λ*_*R*_, as by definition, sensitive cells must be easier for the drug to kill. *f*(*S*, *D*) is the function that represents the transition of sensitive cells to resistant ones (i.e., acquired resistance). This may or may not depend on the drug *D*. *γ* is decay rate of the drug, which we fix using the the fact that the mean half-life of cetuximab is 4.75 days^58^, corresponding to *γ* ≈ 0.1459 days^−1^. Finally, cetuximab administration of 5 mg/kg every 7 days is captured through the initial condition on *D*. In particular, *D*(0) = 5, and every 7 days, *D* is increased by 5 mg/kg to simulate the administration of a new dose.

**Fig. 1:**
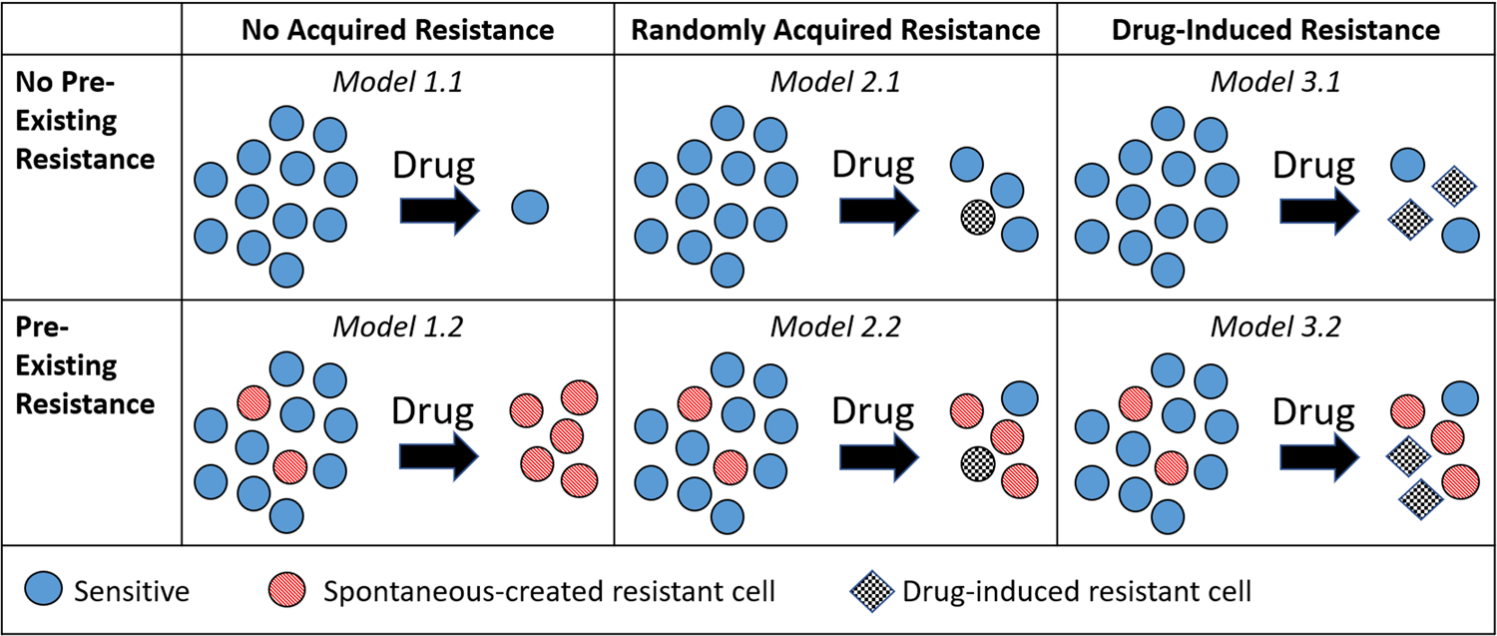
Schematic illustrating the family of resistance models. Sensitive cells are illustrated as blue circles, pre-existing resistant cells as red-striped circles, spontaneously-created resistant cells as black-and-white checkered circles, and drug-induced resistant cells as black-and-white checkered diamonds. Second row contains all models without pre-existing resistance, and the third row contains all models with pre-existing resistance. Second column contains all models with no acquired resistance, third column contains all models with randomly acquired resistance, and fourth column contains all models with drug-induced resistance.

Depending on the assumptions made, this model can represent any combination of: pre-existing resistance (when *R*(0) > 0), randomly acquired resistance (when the transition to resistance *f*(*S*, *D*) is independent of the drug *D*), and drug-induced acquired resistance (when *f*(*S*, *D*) depends on *D*). In all cases, we model resistance as a cellular phenotype regardless of whether they arise from a mutational or transcriptional driver. The different sub-models that we consider are explained here, and visually explained in Fig. 1.

- Model 1: No Acquired Resistance. This requires setting *f*(*S*, *D*) = 0 in Eqs. (7–8). This model can be further broken down into two sub-cases:
  - Model 1.1: No Pre-Existing Resistance. Achieved by setting *R*(0) = 0, meaning the entire tumor population is sensitive to cetuximab.
  - Model 1.2: Pre-Existing Resistance. Achieved by allowing *R*(0) > 0.
- Model 2: Randomly Acquired Resistance. We model this with a random transition term *f*(*S*, *D*) = *f*(*S*) = *gS* in Eqs. (7–8), meaning resistance is independent of drug *D*. This model can be further broken down into two sub-cases:
  - Model 2.1: No Pre-Existing Resistance. Achieved by setting *R*(0) = 0, meaning resistance can only result from the random acquisition of resistance that happen during treatment.
  - Model 2.2: Pre-Existing Resistance. Achieved by allowing *R*(0) > 0, meaning resistance can pre-exist treatment and can be randomly acquired during treatment.
- Model 3: Drug-Induced Acquired Resistance. We model this with a drug-dependent transition term *f*(*S*, *D*) = *gSD* in Eqs. (7–8), as similarly done in ref. ^33^. This model can be further broken down into two sub-cases:
  - Model 3.1: No Pre-Existing Resistance. Achieved by setting *R*(0) = 0, meaning resistance can only result from the drug-induced acquisition of resistance.
  - Model 3.2: Pre-Existing Resistance. Achieved by allowing *R*(0) > 0, meaning resistance can pre-exist treatment and can be induced by the drug during treatment.

### Fitting algorithm

As we have proposed a variety of models to describe resistance of HNSCC to cetuximab, we must determine which model (or models) most accurately describes the experimental data. This requires that we fit each model to the volumetric time-course data of treatment response to cetuximab. Due to the extreme variability between mice, we chose to fit each mouse individually, rather than fit to the average of the time-course data.

For each model, and for each mouse *i*, the parameter set we seek is the one that minimizes the sum of the squared error (SSE), which we will call *ζ*_*i*_:

10 $${\zeta }_{i}=\mathop{\sum }\limits_{t=1}^{{n}_{i}}{({y}_{i}(t)-\bar{{y}_{i}}(t))}^{2},$$

where *y*_*i*_(*t*) represents the experimental tumor volume for mouse *i* at time *t*, and $\bar{{y}_{i}}(t)$ is the tumor volume obtained through the model at time *t*. This is indexed over *n*_*i*_, the number of time points in the data set for mouse *i*.

We implement a two-step fitting approach. The first step uses a Quasi-Monte Carlo (QMC) method to quasi-randomly sample the parameter space. Herein we use Sobol’s low-discrepancy sequences to quasi-randomly sample points across a *k*-dimensional hyperrectangle^59^. *k*-dimensions represents the *k* parameters in the model that are being fit to the data (including the initial condition *S*(0), and *R*(0) in the case of pre-existing resistance). Sobol’ sequences were chosen to sample space as they possess uniformity properties that other sampling techniques lack, have lower discrepancy than other sampling methods for up to dimension 20, while also being more efficient than other sampling methods^59^.

Our algorithm utilizes QMC by first quasi-randomly sampling 1.5 × 10^6^ Sobol points of the form (*p*_1_, …, *p*_*k*_). We chose this number of Sobol points to minimize the computational time required while maximizing coverage of the parameter space. Each _*pi*_ in a sampled point are in the range [0, 1]. We then have to scale the values of _*pi*_ so that they are in a biologically reasonable range for that parameter value. Through numerical exploration, we found it sufficient to scale all non-initial condition and non-carrying capacity parameters except for *r*_*S*_ to be in the range [0, 0.1], as parameter values beyond this result in model predictions of completely different magnitudes than the experimental data. Numerical experimentation suggested optimal *r*_*S*_ values to be greater than 0.1, so this parameter was scaled to be in the range [0, 0.2]. The scaled range for the initial tumor volume was mouse-dependent. The initial condition per mouse were scaled to be in the range [0, 2*V*_0_], where *V*_0_ is the actual initial tumor volume for each mouse (see Supplementary Table 1). Further, the carrying capacity was searched over the range [*V*_0_, 10^5^]. Finally, in the case of the Allee effect, the existential threshold *m* was searched over the range [0, 10*V*_0_].

The model of interest is then solved at the 1.5 × 10^6^ scaled Sobol parameter sets for a given mouse *i*, provided the parameter set is biologically realistic. Biological viability is determined by the restrictions detailed in “Modeling treatment data” based on the fitness disadvantage conferred by drug resistance (*r*_*S*_≥*r*_*R*_ and *λ*_*S*_ > *λ*_*R*_). Each biologically viable parameter set provides us with a cost function value, *ζ*_*i*_(*p*_1_, …, *p*_*k*_). Then, for each mouse *i* we identify the parameter set with the lowest *ζ*_*i*_ value. This parameter set should be close to the optimal parameter set, though generally it is not the actual optimal. The Quasi-Monte Carlo step is summarized in Algorithm 1.

#### Algorithm 1. Quasi-Monte Carlo Method



Once QMC has established an initial “guess” parameter set for each mouse that minimizes *ζ*_*i*_, a simplified version of simulated annealing (gradient descent) is performed to refine the optimal parameter prediction. Simulated annealing is a stochastic optimization method with the goal of finding a global optimum^60^. It begins with an initial set of parameters and evolves the parameters with random perturbations until a specified criteria is met. In particular, for each parameter *p*_*j*_ a random value in the range $[-1{0}^{{\alpha }_{j}-1},1{0}^{{\alpha }_{j}-1}]$ is generated, with ${\alpha }_{j}={{{\mathcal{O}}}}({p}_{j})$ meaning that *α*_*j*_ is the order of magnitude of parameter *p*_*j*_. As an example, if *p*_*j*_ = 0.2 = 2 × 10^−1^, then the order of magnitude is −1 and a random perturbation value is generated in the range [−10^−2^, 10^−2^]. Each new set of randomly perturbed parameters is either accepted or rejected according to the change in *ζ*_*i*_, which is denoted as Δ = ${\bar{\zeta }}_{i}$ - *ζ*_*i*_ where ${\bar{\zeta }}_{i}$ is the SSE of the newly perturbed parameter set and *ζ*_*i*_ is the SSE of last accepted parameter set. If Δ < 0 (i.e., the new SSE is lower), then the change is always accepted, and the new parameter set is saved. As numerical experimentation revealed that accepting uphill parameter changes decreased algorithm performance (likely due to starting “close to” the optimal from the QMC step), we decided not to accept uphill moves, so if Δ > 0, the change not accepted. This algorithm is therefore equivalent to stochastic gradient descent. This perturbation process is repeated 5 × 10^5^ times for each mouse, and the last accepted parameter set with the lowest *ζ*_*i*_ is taken to be the global optimum corresponding to the starting point selected by QMC sampling. The gradient descent procedure is summarized in Algorithm 2.

#### Algorithm 2. Gradient Descent



This two-step algorithm is used in all instances, whether fitting the control or treatment data, with the exception of fitting an exponential curve to the control data as that can be done analytically. In all instances where this numerical fitting algorithm was used, the two-step procedure was repeated 15 times, and for each mouse the parameter set with the lowest SSE of the 15 repetitions was chosen as the optimal parameter set. By implementing this multi-start algorithm (that is, starting at 15 different points in parameter space that are near (typically different) local minima), we improve the likelihood that the algorithm converges to the globally minimum parameter set.

### Identifiability

Identifiability analysis gives us one way to assess the “goodness” of a mathematical model. It is especially relevant in computational models of biological systems given the limited availability and quality (measurement error, noise) of experimental data^61^. Here, we will focus on practical identifiability, analyzing if we have sufficient data to have well-determined values for the model parameters. We use profile likelihood^62^ (without a confidence interval, since these are fits to the individual, not the average) to evaluate the practical identifiability of a particular model parameter: the initial resistance fraction. This is defined by

$${r}_{frac}^{0}=\frac{R(0)}{S(0)+R(0)}.$$

To this end, we define the relevant range for the parameter value, and consider a discrete set of values for the parameter across that range. Since the initial fraction of resistant cells must be in the range [0, 1], we define this range and consider every 0.05 value within this range. At each such ${\bar{r}}_{frac}^{0}$ value, we find the best-fit values of the remaining parameters and plot the optimal value of the cost function *ζ*_*i*_ across the parameter’s range to get a profile likelihood curve. The profile likelihood curve for a practically identifiable parameter should appear quadratic, with a clear minimum at the optimal parameter value. If such a quadratic shape is not achieved, the parameter is not practically identifiable^62^. While this analysis could be performed for all model parameters, we choose to focus on the initial resistant fraction, as it is a parameter that is easy to interpret biologically, and therefore will help us in identifying the most likely model describing the experimental data.

## Results

To select model that “best” describes the mouse data, we utilized two different model selection methods, the Akaike information criterion (AIC):

11 $$AIC={n}_{i}\ln \left(\frac{{\zeta }_{i}}{{n}_{i}}\right)+2k,$$

and the Bayesian information criterion (BIC)^63^:

12 $$BIC={n}_{i}\ln \left(\frac{{\zeta }_{i}}{{n}_{i}}\right)+k\ln ({n}_{i}).$$

In these equations, *n*_*i*_ is the number of data points for mouse *i*, *k* is the number of model parameters to be fit, and *ζ*_*i*_ is the SSE from the optimal parameter set for mouse *i*.

Both information criterion consider the trade-offs between goodness of fit (*ζ*_*i*_) and simplicity of the model (i.e., the number of parameters, *k*), allowing us to compare models with different assumptions. However, these measures penalize the number of parameters differently. The AIC model assumes a penalty of the form 2*k*, which is independent of the number of data points. BIC assumes the penalty term $k\ln ({n}_{i})$, meaning the weight of the parameter penalty increases as the number of data points increases. The more data points there are, the greater this penalty term is for the BIC as opposed to the AIC. The model with the lowest AIC (or BIC) score is considered to be the most parsimonious, meaning it achieves reasonable fits the data using a minimal number of parameters. We will use these two information criterion as we compare our proposed models for the control and treatment data.

### Selecting a model: control data

**Fig. 2:**
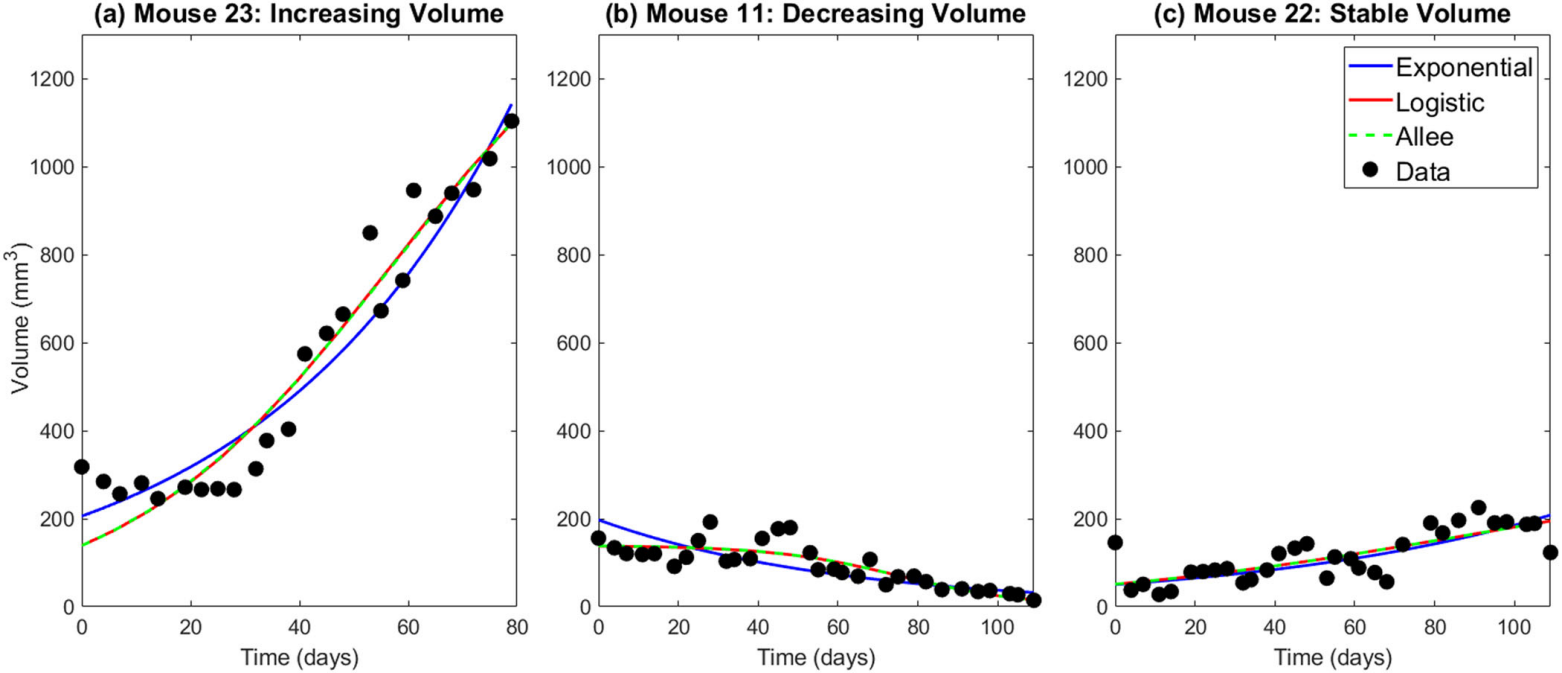
Best fit exponential, logistic, and Allee model for three representative mice. **a** Mouse 23 representing the case where the tumor volume increases. **b** Mouse 11 representing the case where the tumor volume decreases. **c** Mouse 22 representing the case where the tumor volume remains relatively stable.

In “Modeling control data”, we proposed three models to fit the control data: exponential, logistic, and Allee. Mouse 23, 11 and 22 each show different tumor growth behavior of increasing in volume, decreasing in volume, or stabilized volume, respectively. Therefore, we use these three mice in Fig. 2 as representatives to visualize the goodness-of-fit of the various models to the experimental data.

**Fig. 3:**
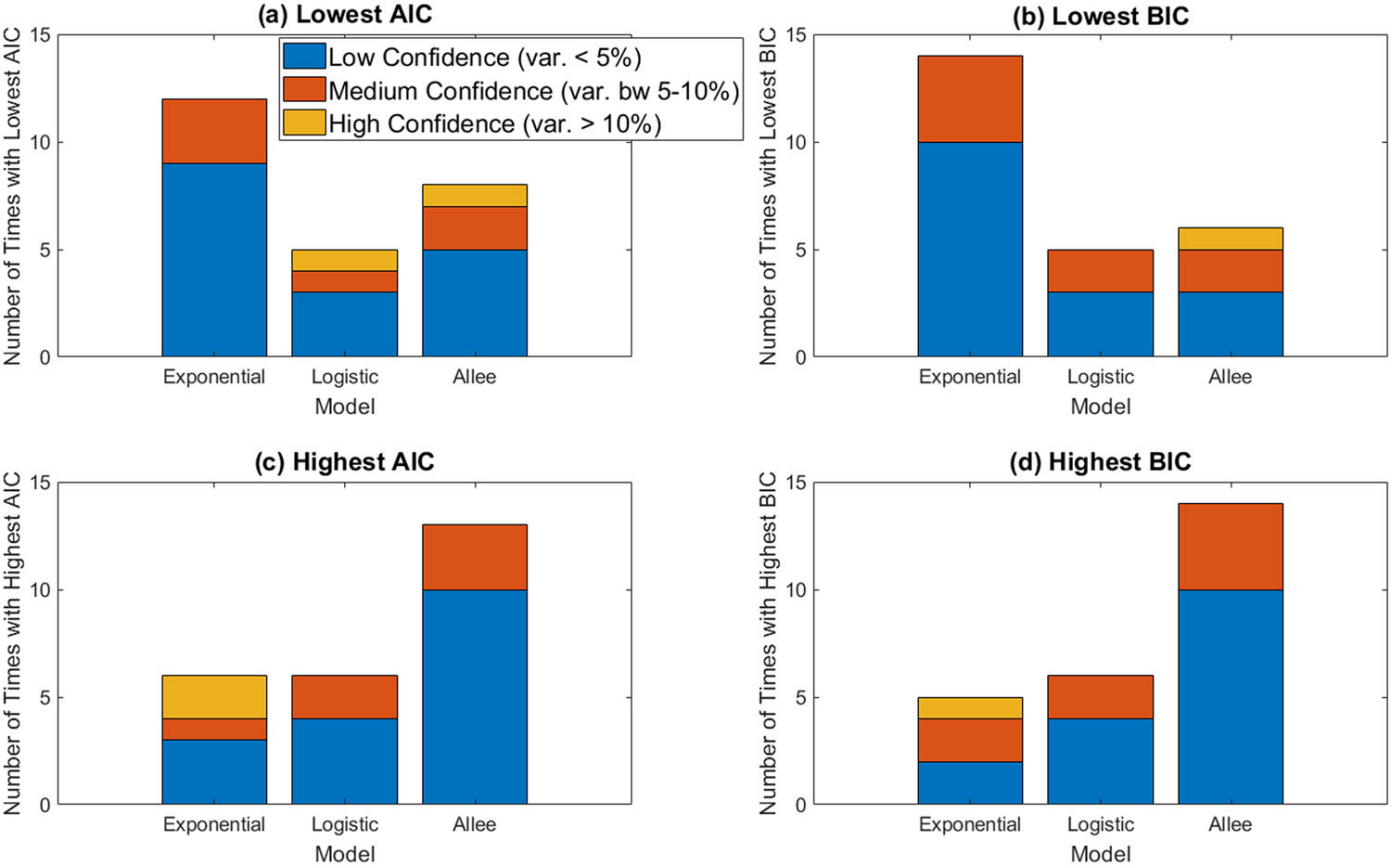
AIC and BIC comparisons across control models. **a**, **b** The number of mice for which each model has the lowest IC value (i.e., is the most parsimonious model), with (**a**) using AIC and (**b**) using BIC. **c**, **d** The number of mice for which each model has the highest IC value (i.e., is the least parsimonious model), with (**c**) using AIC and (**d**) using BIC. We have low confidence in our classification (blue) when the IC value corresponding to the most parsimonious (or least parsimonious, for high IC) model varies by 5% or less from the other IC values. We have medium confidence (red) when it varies by 5–10%, and high confidence (yellow-orange) when it varies by >10%.

AIC and BIC values are used for model selection, where the lower the IC value, the more parsimonious the model is with the data. As shown in Fig. 3a, b, the exponential model appears to be the most parsimonious model, as it has the lowest AIC in 12 of 25 control mice. Besides asking which model has the lowest AIC, we also ask: how much lower is it than the AIC for the next-best model? If the AIC for the top model is at least 15% smaller than the AIC of the next-best model, we classify this as “high confidence” - in other words, we have high confidence that the model with the lowest AIC is the most parsimonious model for describing the data. If the AIC for the top model is within 5% of the AIC for the next-best model, we call this “low confidence”. The remaining case gets classified as “medium confidence.” As seen in Fig. 3, we found that in 9 of the 12 control mice for which the exponential model has the lowest AIC, the prediction that the exponential model is most parsimonious is made with “low confidence”. The 3 remaining mice for which the exponential model gives the lowest AIC are classified as “medium confidence”. The trends are very similar if we use BIC instead. The exponential has the lowest BIC (and is thus the most parsimonious model) for 14 of the 25 control mice. We have “low confidence” in this prediction for 10 of 13 mice in which exponential has the lowest BIC and “medium confidence” for the remaining 4 mice.

Despite this preference for the exponential model according to both the AIC and BIC, caution is warranted. Selecting a model by minimizing the IC would result in choosing the Allee differential equation for 8 of the mice according to AIC, and 6 of the mice according to BIC. Given the ambiguities in selecting the model for control growth when looking at the lowest IC values, we also looked at the breakdown of which model is the “worst” (least parsimonious) for describing the data across the control mice; that is, we look for models with the highest IC values. As shown in Fig. 3c, d, the Allee model has the highest IC value in the majority of mice (in 13 of 25 mice according to the AIC, and 14 of 25 mice according to the BIC). Compare this to exponential growth, where the IC is rarely the largest (this occurs in 6 of 25 mice using AIC and 5 of 25 mice using BIC). Considering how often the exponential is the most parsimonious option of the three (as defined by having the lowest IC) and how rarely it is the least parsimonious (as defined by having the highest IC) we will proceed by using an exponential growth term in the treatment models. To assess the robustness, we also consider how our predictions change if we used logistic growth instead.

While the above analysis was limited to three empirical non-spatial models of control tumor growth, it can be readily extended to include other models. Two interesting models to consider are the surface and the von Bertalanffy equation, as they were both developed to mimic the spatial growth of three dimensional tumors using a single, non-spatial, ordinary differential equation. The surface equation $\dot{V}=aV{(V+b)}^{-1/3}$ can be used to describe the change in the tumor volume *V* under the assumption that only a thin layer of cells at the surface are capable of proliferating^37^, whereas the von Bertalanffy equation $\dot{V}=a{V}^{2/3}-bV$ assumes growth occurs at a rate proportional to the surface area, and also accounts for a decrease in tumor volume due to cell death^37,38^. Even though we lack the data to parameterize a spatial model, these ODE models have the same number of parameters as the logistic ODE, and therefore are logical extensions to include in our family of control models. Interestingly, we found that if we expanded our family of models to include these spatially-motivated ODEs, both AIC and BIC still select exponential growth as most parsimonious with the data (see Supplementary Fig. 1).

### Insufficiency of volumetric data for treatment model selection

**Fig. 4:**
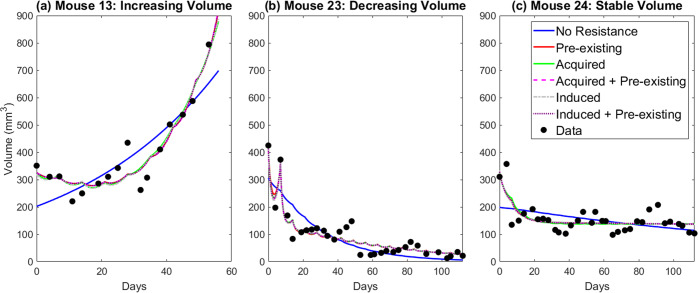
Best fit of six proposed resistance models to treatment data for three representative mice. **a** Mouse 13 representing the case where the tumor volume increases in spite of treatment. **b** Mouse 23 representing the case where the tumor volume decreases during treatment. **c** Mouse 24 representing the case where the tumor volume remains relatively stable during treatment.

In “Modeling treatment data”, we proposed a family of six models to describe the resistance of cetuximab in our xenograft data (see Fig. 1): Model 1.1 with no resistance, Model 1.2 with pre-existing resistance only, Model 2.1 with randomly-acquired resistance only, Model 2.2 with randomly-acquired and pre-existing resistance, Model 3.1 with drug-induced resistance only, and Model 3.2 with drug-induced and pre-existing resistance. Assuming exponential growth as justified in “Selecting a model: control data”, the fits of the six treatment models to data for three representative mice that exhibit different tumor growth dynamics are shown in Fig. 4. The best-fit value of all parameters, across all mice and models, is shown in Supplementary Fig. 3.

**Fig. 5:**
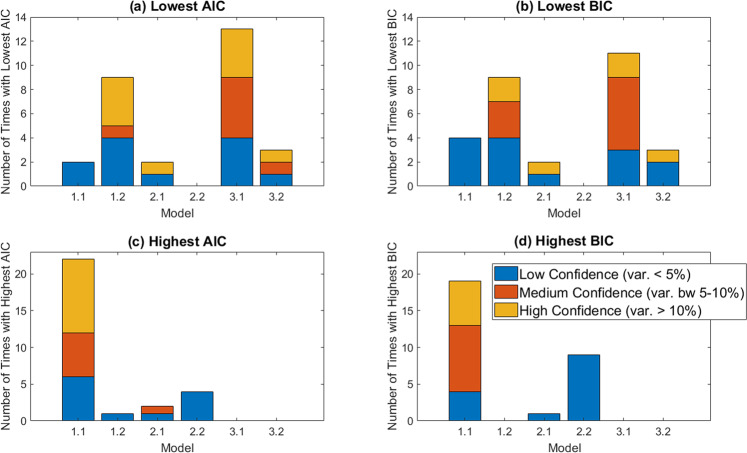
AIC and BIC comparisons across treatment models when exponential growth is used. **a**, **b** The number of mice for which each model has the lowest IC value (i.e., is the most parsimonious model), with (**a**) using AIC and (**b**) using BIC. **c**, **d** The number of mice for which each model has the highest IC value (i.e., is the least parsimonious model), with (**c**) using AIC and (**d**) using BIC. Confidence in model selection is also shown, as described in detail in Fig. 3.

A visual inspection of the fits suggests that Model 1.1 (no resistance) cannot adequately explain treatment response to cetuximab. In order to quantitatively approach model selection so as to determine which “member(s)” of our family of resistance models most likely captures the mechanisms in the data, we computed the AIC and BIC for each mouse and model (Fig. 5). This analysis confirms that some form of resistance must be driving treatment response to cetuximab, as Model 1.1 very rarely has the lowest IC value (happens 2 of 29 times for AIC, and 4 of 29 times for BIC), and very frequently has the highest IC value (happens 22 of 29 times for AIC, and 19 of 29 times for BIC). By a similar argument, the IC values indicate that the resistance in the data likely cannot be attributed to a combination of randomly acquired and pre-existing resistance (Model 2.2). As indicated in Fig. 5, Model 2.2 never has the lowest IC value, and occasionally has the highest IC value (happens 4 of 29 times for AIC, and 9 of 29 times for BIC). Therefore, this information theoretic analysis was able to rule out several mechanistic explanations of cetuximab resistance, but it is not sufficient to select the model whose mechanisms most likely explain this resistance. Notably, the case of no resistance, and the case of resistance being pre-existing and randomly-acquired, are also ruled out if growth is assumed to be logistic instead of exponential (see Supplementary Fig. 4).

### Initial pre-existing resistance fraction facilitates treatment model selection

Our analyses thus far assume that the only available data is the time-course describing tumor volume in individual xenografts. Advances in single-cell profiling technologies^64^ can now quantify the initial fraction of cells with specific therapeutic resistance mechanisms. While such analyses were not undertaken for the xenograft data presented herein, our modeling framework could be readily used to ask: does the inclusion of the initial resistance fraction improve our model selection capabilities?

Before we explore this question in depth, we first consider each model’s prediction regarding the initial resistance fraction. Model 1.2 has a median resistance fraction of 53.01%, with a mean of 57.18 ± 27.28%. Model 2.2 has a median resistance fraction of 25.55%, with a mean of 35.96 ± 28.80%. Model 3.2 has a median initial resistance fraction of 10.48%, with a mean of 31.75 ± 38.06%. While our previous studies have identified molecular mechanisms of cetuximab resistance expressed before resistance develops in cetuximab sensitive HNSCC cell lines^13^, we note that these proportions of initially resistant cells exceed those of our previous studies, and seem large when considering that resistance often results in a fitness disadvantage in the absence of drug^55,56^. In this section, we further explore how the value of the initial resistance fraction contributes to model fits. Given that evidence in ref. ^13^ supports the presence of some pre-existing resistant cells, herein we will only consider models that include pre-existing resistance.

**Fig. 6:**
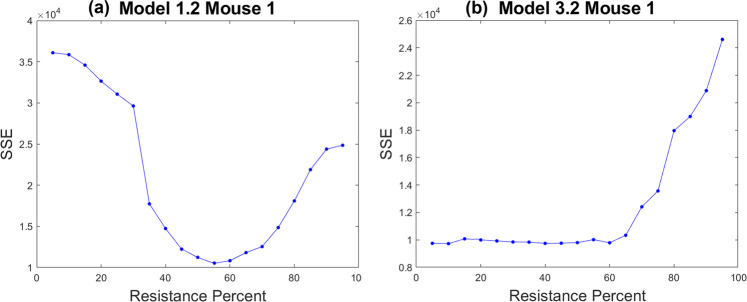
Profile likelihood curves of the initial resistance fraction for a representative mouse, Mouse 1. **a** The resistance fraction in Model 1.2 is practically identifiable and places the optimal parameter at approximately 55%. **b** The resistance fraction in Model 3.2 is not practically identifiable.

We will study the contribution of the initial resistance fraction to model fits by determining the practical identifiability of this parameter using the profile likelihood method. We consider Model 1.2 (pre-existing resistance only) and Model 3.2 (drug-induced acquired plus pre-existing resistance), though not Model 2.2 as our information theoretic analysis already concluded that the combination of randomly acquired and pre-existing resistance is a highly unlikely to explain cetuximab resistance in our xenograft data. In 20 of 29 mice fit using Model 1.2, the profile likelihood curves for the initial resistance fraction reveal this parameter to be practically identifiable. The profile likelihood curve for Mouse 1 in Fig. 6a is representative of these 20 mice. For Mouse 1, we observe a clear optimal value for the initial resistance at ${r}_{frac}^{0}\approx 0.55$. Any significant deviation from this resistance fraction drastically increases the cost function (that is, decreases the goodness-of-fit). In this case, having the true value of the initial resistance fraction could greatly inform the process of model selection. If the true initial resistance fraction was ${r}_{frac}^{0}=0.05$, the cost function increases more than three-fold. This in turn increases the IC values in Eqs. (11) and (12), and significantly reduces the likelihood of Model 1.2 having the lowest IC values. In other words, this would provide strong evidence that pre-existing resistance alone does not explain cetuximab resistance in the data.

Compare this to what happens in the same mouse fit using Model 3.2 (pre-existing plus drug-induced resistance). The profile likelihood curve for the same parameter is not practically identifiable, as demonstrated in Fig. 6b by a shallow profile with a one-sided minimum. This is not unique to Mouse 1 - the profile likelihood curves for the initial resistance fraction in Model 3.2 reveal this parameter to be practically non-identifiable in 22 of 29 mice. Returning to our prior thought experiment, for Mouse 1 in particular, if we had measured the initial resistance fraction to be ${r}_{frac}^{0}=0.05$, we would have strong evidence that Model 3.2 should be selected over Model 1.2. While the lack of practical identifiability poses mathematical challenges, it does give Model 3.2 a lot more “flexibility” to conform to additional experimental data without sacrificing goodness-of-fit.

Finally, it is important to note that incorporating such single-cell data into our model does require representative sampling of the entire cellular population, and having prior knowledge of a marker of drug resistance. Even if this data were available, the addition of the initial resistance fraction is not always sufficient to select a model. Continuing to use Mouse 1 as an example, if we had measured the true initial resistance fraction to be ${r}_{frac}^{0}=0.55$, both Models 1.2 and 3.2 remain viable choices. Therefore we conclude that while measuring the initial resistance fraction is an essential step to understanding the mechanisms underlying cetuximab resistance, it is not guaranteed to determine the mechanism of resistance using our family of mathematical models.

### Dose escalation study further facilitates treatment model section

**Fig. 7:**
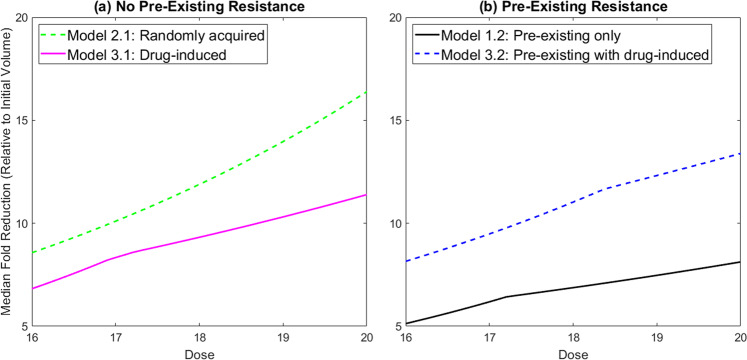
Dose escalation study of median reduction in tumor volume (relative to initial volume) after 2 weeks. Dose varies from 16 to 20 mg/kg. Growth is assumed to be exponential. **a** Plausible models involving no pre-existing resistance. **b** Plausible models including pre-existing resistance.

Thus far we have established that time-course volumetric data combined with a measurement of the initial resistance fraction may or may not be sufficient to deduce the underlying mechanism of resistance using our family of models. Here, we propose a final experiment that, combined with the other data, would be sufficient to select a treatment model. In particular, we propose a dose escalation study where we use the optimal parameter set for each mouse to simulate tumor response to a range of drug doses. We measure the fold reduction in the tumor volume per mouse by comparing the initial tumor volume in each mouse to its volume 2 weeks later, with one dose of cetuximab given per week as in the experimental protocol. The median fold reduction across all 29 mice, at each dose, is then computed (see Fig. 7). As an example, a median fold reduction of 4 means the median tumor volume is four times smaller post-treatment than it was pre-treatment. Thus a higher median fold reduction represents a more effective treatment.

We computationally experimented with different dose ranges and time frames for this dose escalation study. We ended up selecting a dosing range of 16–20 mg/kg, and found that weekly dosing over 2 weeks allows us to distinguish the behavior of the different models. Figure 7a shows the dose escalation results in the case of no pre-existing resistance. Starting at a dose of 16 mg/kg, simulations show a more significant fold reduction in median tumor volume when resistance is randomly acquired (8.571 median fold reduction) than when resistance is drug-induced (6.822 median fold reduction). The lower response in the drug-induced case can be explained by the fact that the transition from the sensitive to the resistant phenotype is directly promoted by the drug itself. Thus higher drug doses drive more resistance formation in the drug-induced case, though not in the randomly acquired case.

Looking across doses, we also observe a noticeably different change in the median volumetric fold-reduction as the dose is escalated from 16 mg/kg to 20 mg/kg. The average rate of change in the randomly acquired case is 1.955, whereas the average rate of change in the drug-induced case is only 1.143. This strongly suggests that one way to distinguish between modes of acquired resistance, at least in the absence of pre-existing resistance, is to experimentally perform this dose escalation study.

Figure 7b shows the two plausible models in the case of pre-existing resistance. Focusing on the dose of 16 mg/kg, simulations show a more significant fold reduction in median tumor volume when resistance can be induced by the drug. This occurs because the initial resistance fraction required to fit the data when the model does not include drug-induced resistance is necessarily larger than the initial resistance fraction when there is a secondary mechanism for creating resistant cells (in the case of Model 3.2, the drug itself promotes the transition to resistance). Therefore, Model 1.2 mice always have a larger initial resistance fraction, and thus experience a smaller response to the drug over the relatively short time period of 2 weeks. However we observe a more significant change in the median fold reduction in the case of drug-induced resistance as the dose is escalated from 16 mg/kg to 20 mg/kg. In particular, the average rate of change in the drug-induced case is 1.309, whereas the average rate of change in the case where all resistance is pre-existing is 0.749. Whether resistance is pre-existing or not, we see an approximately 1.7-fold difference in the median across models, demonstrating that an experimental dose escalation study would provide meaningful data in trying to elucidate the mechanisms driving cetuximab resistance.

## Discussion

In this work, we introduced a family of six ordinary differential equation models, with each model assuming a different underlying biological mechanism(s) driving cetuximab resistance in patient-derived xenografts of head and neck squamous cell carcinoma. Model selection techniques alone allowed us to conclude that some form of resistance must be driving the treatment response dynamics, and that this resistance was highly unlikely to be explained by randomly acquired resistance coupled with pre-existing resistance.

With four family members remaining to plausibly describe cetuximab resistance, we next asked: what additional data would be needed so that we can identify the model from the family that is most parsimonious with the data? Through the use of profile likelihood curves, we uncover that quantifying the initial fraction of resistant cells in a tumor population improves the likelihood of identifying the model within the family that is most parsimonious with the data. Therefore, we hypothesize that integrating single-cell data with these mathematical models can be used to predict resistance mechanisms in vivo, building on foundational work integrating these data into in vitro mathematical models of drug resistance^65^. Even in absence of molecular profiling data (though requiring an assumption on whether there are pre-existing resistant cells or not), we find volumetric data from dose escalation studies would enable our mathematical modeling approach to distinguish between the various mechanisms potentially causing cetuximab resistance.

The conclusions drawn in this work are dependent on the family of models constructed. While we have demonstrated some robustness in the results to the underlying growth term in the absence of treatment, other assumptions and functional forms for both tumor growth and drug effects could certainly be considered. As future work, one option would be to expand the family of models and repeating the analyses herein to determine if a combination of time-course volumetric data at a single dose, measurements of the initial resistance fraction, and dose escalation data are sufficient experimental data to pinpoint the mechanism and timing of resistance. This could include analyzing the same models under the assumption that resistance does not incur a fitness disadvantage^57^, and can also include adding models to the family (both spatial and non-spatial) that are more mechanistic in nature^52^. While growing the number of models considered in the family is one approach for future work, an alternative approach is to use model learning techniques^66–73^, possibly informed by biological knowledge^74^. Model learning provide tools for considering a much larger class of mathematical models of cetuximab resistance in HNSCC, and uses the available data to “learn” the underlying dynamical system describing the data.

Beyond considering a larger family of models, in the future we can also consider different approaches for identifying model parameters. Herein, we approached fitting each mouse independently, even though the mice are part of the same population. Nonlinear mixed effects models provide a compromise between fitting the average and fitting the individual, by incorporating both fixed effects (population-level parameters) and random effects (parameters that differ between individuals in the population) in the regression problem^75^. Another option would be to consider a nonparametric fitting methodology, for instance using Bayesian inference. In this case, parameters are assumed to be random variables with unknown probability (posterior) distributions that quantify the likelihood of the parameter assuming any value in the parameter space^76^. Given the variability in the individual mice data, a Bayesian approach would provide uncertainty quantification, and help understand correlations between model parameters.

Understanding the mechanisms driving cetuximab resistance is essential, as optimal therapeutic design is likely dependent on the underlying mode(s) of resistance. For instance, work in ref. ^33^ computationally demonstrated that tumor response to the same drug dose and delivery schedule is qualitatively impacted by the ability (or lack therefore) of a drug to induce resistance. Therefore it is essential that any mathematical model accurately capture the mechanism driving resistance if that model is to be used to optimize drug dosing and the delivery schedule. Although the data-driven approach in this work did not allow the mechanism(s) driving cetuximab resistance to be uncovered, it did lead to an experimental design that we propose would provide the data needed to elucidate the underlying resistance mechanisms. Understanding the generality of these mechanisms to cetuximab resistance in HNSCC and their broader applicablity to targeted therapeutic resistance requires further evaluation in additional xenograft models. Therefore, in future work, we will perform follow-up studies to validate (and possibly refine) the proposed experimental design. This will enable us to draw conclusions about the mechanisms that drive cetuximab resistance, which in turn will inform optimal dosing strategies.

## Supplementary information

Supplemental Material

## Data Availability

All experimental data are publicly available at https://github.com/jgevertz/HNSCC-Cetuximab-Resistance.

## Code availability

All fitting codes are publicly available at https://github.com/jgevertz/HNSCC-Cetuximab-Resistance.
