# Supplementary material for: Model-informed experimental design recommendations for distinguishing intrinsic and acquired targeted therapeutic resistance in head and neck cancer
Source: NPJ Syst Biol Appl. 2022 Sep 8;8:32. doi: 10.1038/s41540-022-00244-7 (PMC9458753; doi:10.1038/s41540-022-00244-7)
Supplement: Supplementary file 1 — Supplemental Material [file 41540_2022_244_MOESM1_ESM.pdf]

Model-informed experimental design recommendations for distinguishing intrinsic and acquired targeted therapeutic resistance in head and neck cancer

Santiago Cárdenas, Constance J. Reznik, Ruchira Ranaweera, Feifei Song, Christine H. Chung, Elana J. Fertig*, Jana L. Gevertz*

Supplementary Information

Mouse ID	Initial Control Volume (mm^3)	Initial Treatment Volume (mm^3)
**1**	150	538.28
**2**	473.8	298.58
**3**	549.5	159.83
**4**	596.8	288.71
**5**	346.4	871.67
**6**	522.6	501.84
**7**	571.8	105.47
**8**	1008.4	279.4
**9**	442	410.60
**10**	628.6	279.20
**11**	155.9	547.97
**12**	339.8	205.27
**13**	624.4	350.98
**14**	1073.2	616.01
**15**	559.4	1188.23
**16**	281	524.62
**17**	727.8	935.25
**18**	410.6	391.13
**19**	550.1	551.35
**20**	422.2	1274.23
**21**	909.6	452.07
**22**	145.8	348.55
**23**	317.7	424.87
**24**	228.3	310.44
**25**	724.9	744.675
**26**	–	397.37
**27**	–	1111.57
**28**	–	1097.87
**29**	–	842.41

Supplementary Table 1: Volume measurements (mm^3) for the 25 control mice and 29 treatment mice. Note that Mouse m in the control case is not related to Mouse m in the treatment case.

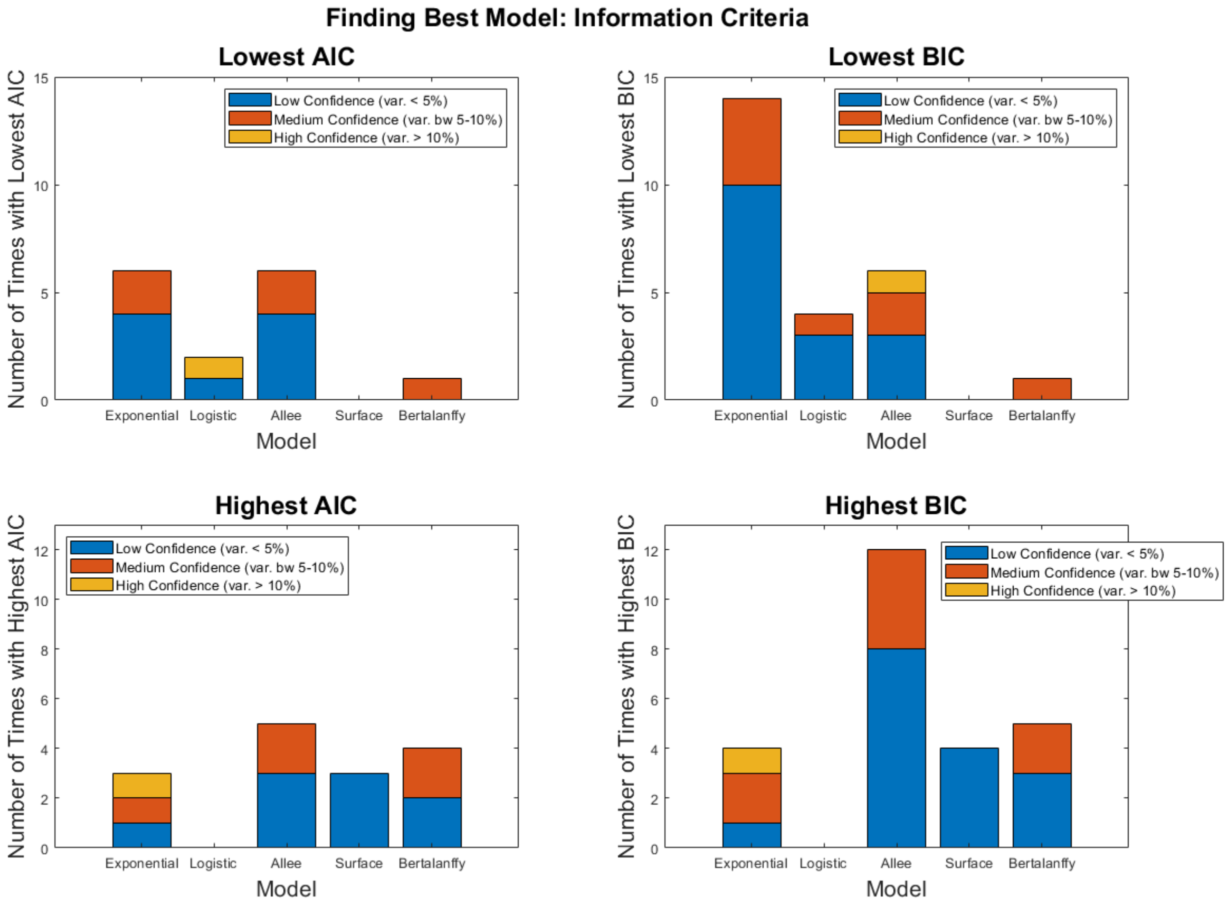

Supplementary Figure 1: AIC (left column) and BIC (right column) comparisons across control models, with the family extended to include the surface ($\dot{V} = aV(V+b)^{-1/3}$) and von Bertalanffy ($\dot{V} = aV^{2/3} - bV$) ODEs. For QMC sampling for the surface model, $a \in [-1, 2]$ whereas the range for b was determined the same way as the carrying capacity in the logistic and Allee ODE. For QMC sampling for the von Bertalanffy model, $a \in [0, 2]$ and $b \in [0, 1]$. Top row shows the number of mice for which each model has the lowest IC value (i.e., is the most parsimonious model). Bottom row shows the number of mice for which each model has the highest IC value (i.e., is the least parsimonious model). Confidence in model selection is also shown, as described in detail in Fig. 3.

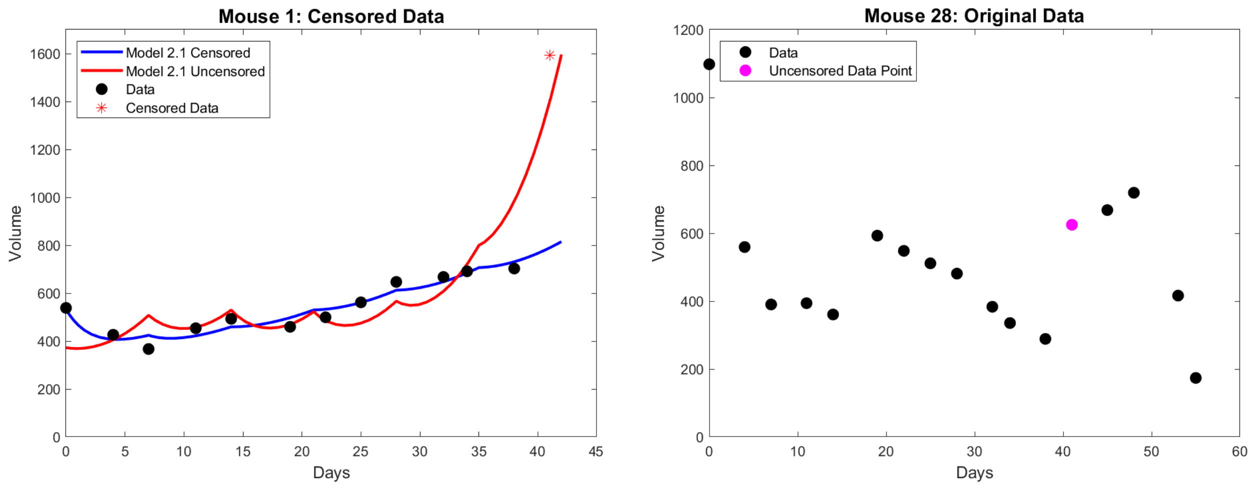

Supplementary Figure 2: Two representative treatment mice to depict censoring protocol. Uncensored data for Mouse 1 (left panel) is shown in black dots with the censored point as a red asterisk. The best-fit curve from Model 1.2 on both censored and uncensored data is shown. Mouse 28 (right panel) had no censored data points. The magenta data point is candidate for censoring because of the rapid growth over a short period of time, but it is not censored because the subsequent points follow the trend.

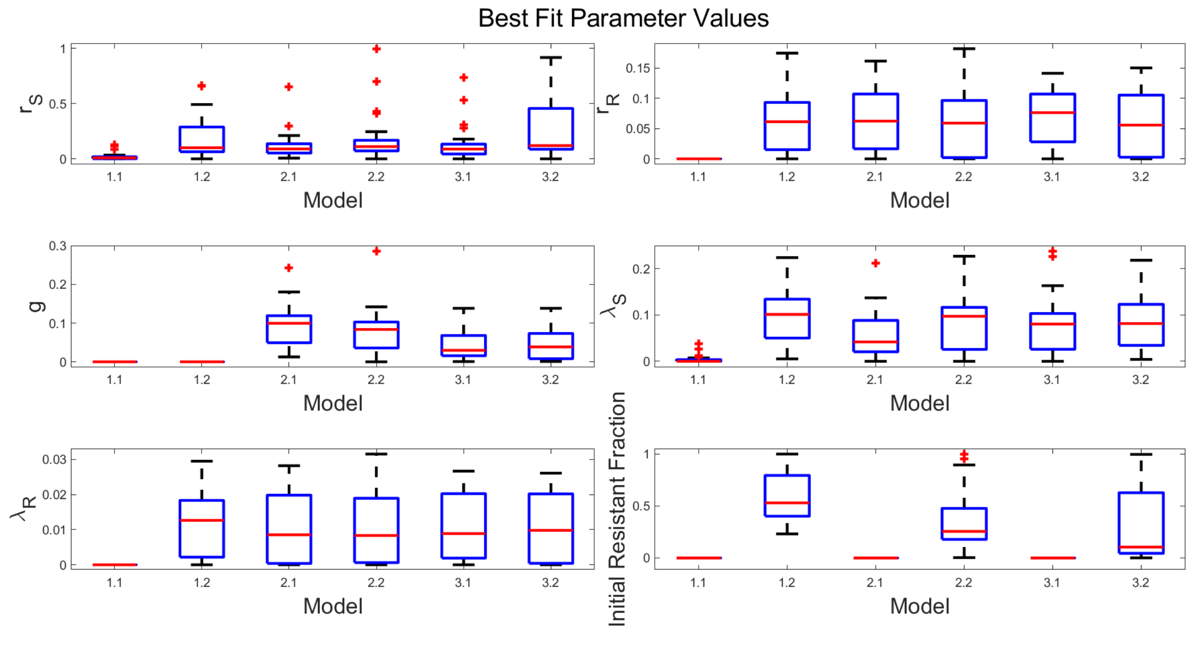

Supplementary Figure 3: Box plot showing best-fit parameter values across six models for sensitive cell growth rate r_S (top left), resistant cell growth rate r_R (top right), rate of transition to resistance g (center left), drug-induced death rate of sensitive cells λ_S (center right), drug-induced death rate of resistant cells λ_R (bottom left), and initial resistant fraction $r_{frac}^0 = R(0)/(S(0) + R(0))$ (bottom right).

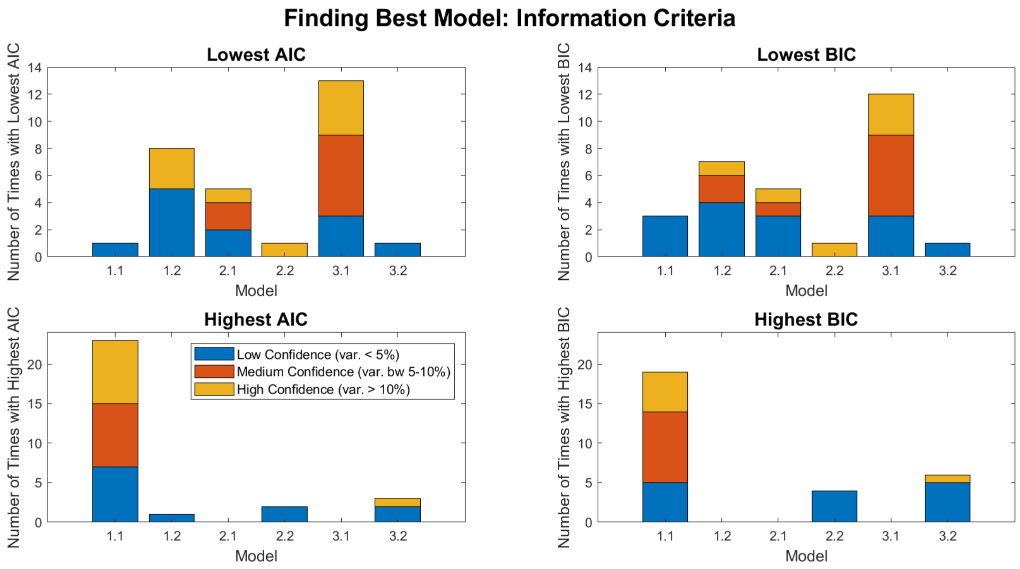

Supplementary Figure 4: AIC (left column) and BIC (right column) comparisons across treatment models when logistic growth is used. Top row shows the number of mice for which each model has the lowest IC value (i.e., is the most parsimonious model). Bottom row shows the number of mice for which each model has the highest IC value (i.e., is the least parsimonious model). Confidence in model selection is also shown, as described in detail in Fig. 3.
